# Penicillin treatment for patients with Community-Acquired Pneumonia in Denmark: a retrospective cohort study

**DOI:** 10.1186/s12890-017-0404-8

**Published:** 2017-04-20

**Authors:** Gertrud Baunbæk Egelund, Andreas Vestergaard Jensen, Stine Bang Andersen, Pelle Trier Petersen, Bjarne Ørskov Lindhardt, Christian von Plessen, Gernot Rohde, Pernille Ravn

**Affiliations:** 1 Department of Pulmonary and Infectious Diseases, Nordsjaellands Hospital, Dyrehavevej 29, 3400 Hillerød, Denmark; 2 Department of Infectious Diseases, Hvidovre Hospital, Kettegård Allé 30, 2650 Hvidovre, Denmark; 3 Center for Quality, Region of Southern Denmark, P.V. Tuxensvej 3-5, 5500 Middelfart, Denmark; 4 Institute for Regional Health Research, Faculty of Health, University of Southern Denmark, Winsløwparken 19, 3, 5000 Odense C, Denmark; 5 Department of Respiratory Medicine, Maastricht University Medical Center, P.O. Box 5800, 6202AZ Maastricht, Netherlands; 6 CAPNETZ-Stiftung, Hannover Medical School, Carl-Neuberg-Str. 1, 30625 Hannover, Germany; 7 University of Copenhagen, Faculty of Health and Medical Sciences, Blegdamsvej 3B, 2200 Copenhagen, Denmark

**Keywords:** Community-acquired pneumonia, Incidence, Penicillin, Prognosis

## Abstract

**Background:**

Community-acquired pneumonia (CAP) is a severe infection, with high mortality. Antibiotic strategies for CAP differ across Europe.

The objective of the study was to describe the epidemiology of CAP in Denmark and evaluate the prognosis of patients empirically treated with penicillin-G/V monotherapy.

**Methods:**

Retrospective cohort study including hospitalized patients with x-ray confirmed CAP. We calculated the population-based incidence, reviewed types of empiric antibiotics and duration of antibiotic treatment. We evaluated the association between mortality and treatment with empiric penicillin-G/V using logistic regression analysis.

**Results:**

We included 1320 patients. The incidence of hospitalized CAP was 3.1/1000 inhabitants. Median age was 71 years (IQR; 58–81) and in-hospital mortality was 8%. Median duration of antibiotic treatment was 10 days (IQR; 8–12). In total 45% were treated with penicillin-G/V as empiric monotherapy and they did not have a higher mortality compared to patients treated with broader-spectrum antibiotics (OR 0.92, CI 95% 0.55–1.53).

**Conclusion:**

The duration of treatment exceeded recommendations in European guidelines. Empiric monotherapy with penicillin-G/V was commonly used and not associated with increased mortality in patients with mild to moderate pneumonia. Our results are in agreement with current conservative antibiotic strategy as outlined in the Danish guidelines.

## Background

Community-Acquired Pneumonia (CAP) is the most frequent lethal infection in Europe, and a substantial economic problem, mainly due to frequent hospital admission [1–3]. Reported incidence ranges from 1.1 to 8:1000 in different studies [4–6]. Lower respiratory tract infections (LRTI), including CAP, are the most frequent indications for prescribing antibiotics [7]. Due to the difficulties of obtaining a microbiological diagnosis, empirical treatment is far more common than pathogen specific treatment [1]. In Denmark, as in other Scandinavian countries and the Netherlands [5, 6], empirical treatment with penicillin-G/V alone is recommended for patients with a CURB-65 score < 3 and combination therapy for patients with CURB-65 ≥ 3 (http://www.infmed.dk/guidelines). Meanwhile, European and American guidelines recommend combination therapy for all hospitalized patients [7, 8]. To which extent monotherapy with penicillin G/V is used and whether this approach is associated with a poorer prognosis is sparsely documented. Moreover, there is a need to investigate the duration of treatment because of the risk of microbiological resistance, side-effects and higher costs that are associated with long treatment duration. Current European guidelines recommend 5–7 days in a responding patient [7], while Danish guidelines recommend 7 days antibiotic treatment for patients with CURB-65 < 3 and 10–14 days for patients with CURB-65 ≥ 3 (http://www.infmed.dk/guidelines).

Thus, in this study, we describe the epidemiology and characteristics of patients hospitalized with CAP in Denmark. Moreover, we investigate the duration of antibiotic treatment and the association between use of penicillin G/V and prognosis.

## Methods

### Design, setting and population

We conducted a retrospective cohort study including all patients admitted with CAP to one large regional and two smaller local hospitals in North Zealand, Denmark. The study period was January 1^st^ 2011 until June 30^th^ 2012. The number of cases hospitalized during the study period determined the sample size. The three hospitals supply acute care for 360,000 inhabitants in the region with urban and rural areas and a socio-demographically varied population. Denmark has universal health insurance that covers all acute care including hospital admissions.

Patients admitted with pneumonia were identified by ICD-10 codes registered at discharge. All patients who had one of the following ICD-10 codes either as primary or secondary diagnosis were considered for inclusion: pneumonia J10.0, J11.0, J12.X – J18.x and J69.X, *mycoplasma* B96.0, *klebsiella* B96.1, *ornithosis* A70.X and *legionellosis* A481.

We reviewed all patient files to assess whether inclusion and exclusion criteria were met. Each patient could only be included once in the study.

The inclusion criteria were: Adult patient’s ≥ 18 years admitted to hospital with CAP. CAP was defined as a new infiltrate on the chest X-ray assessed by the radiologist on-call and at least one of the following symptoms of LRTI: cough, purulent expectoration, fever (≥38.3 °C rectally or ≥37.8 °C auricular) or pathological lung auscultation. Only in-patients were included in the study.

The exclusion criteria were: hospital admission during the last 28 days, active tuberculosis or immunosuppression. Patients were classified as immunosuppressed if they had received treatment with corticosteroids (≥ 20 mg prednisolone-equivalent/day > 14 days), were HIV-positive, had received cancer-chemotherapy during the last 28 days and had neutropenia (neutrophil granulocytes < 1000/μl) or were immunosuppressed after an organ transplantation.

Patients admitted from nursing home and patients with frequent healthcare contacts were considered as having acquired pneumonia in the community [9, 10] and were not excluded from the cohort.

### Data collection and variables

We registered data into the CAPNETZ database (www.capnetz.de) and into a local database in EpiData entry 3.1 (www.epidata.com). Data collection was performed from September 2014 until January 2015. All data stem from electronic patient files as well as laboratory, microbiological and radiological databases.

For each patient, we recorded demographics, comorbidities, symptoms, clinical values including CURB-65 [11], and biochemical test results on admission. Patients were divided in to risk groups according to CURB-65 score (score 0–1, 2 or 3–5). The number of co-morbidities was assessed by categorizing patients into three groups based on the sum of conditions (none, one, and more than one). In addition, we recorded microbiological test results, complications and the length of the hospital admission (LOS). We registered the total duration of antibiotic treatment (LOAB) and duration of intravenous antibiotic treatment (LOIVAB). Only the therapeutic agents given initially were recorded. We followed patients for 6 months after admission, to register deaths. Variables not mentioned in the patient file were noted as missing, except for co-morbidities which were recorded as absent if not mentioned.

### Outcome measures, exposures and confounders

The main outcomes were in-hospital (short term) and 90-day (long term) mortality. Both outcomes were registered from electronic patient files, which also contain information on deaths outside of hospital. Patients were grouped according to empirical antibiotic treatment, which was the main exposure evaluated in this study. Patients who received empirical treatment with monotherapy penicillin-G/V were compared with patients receiving empirical treatment with all other antibiotics, as monotherapy or in combination. CURB-65 score, number of co-morbidities (0, 1 or >1), admission to the ICU, and age were viewed as potential confounders.

Confounding by indication could influence the results, which was addressed by adjusting for confounders, especially CURB-65 score. Further, we stratified patients by CURB-65 score and compared the two treatment groups within risk groups.

### Statistics

We report descriptive statistics at baseline as counts (%) and as mean with standard deviation (SD) or medians with 25^th^ to 75^th^ interquartile range (IQR). When patients died in hospital, we recorded LOS as missing. For patients who died while on antibiotic treatment, we recorded LOIVAB and LOAB as missing values. Group comparisons between the two treatment groups and subgroup analysis were performed with chi-square test for categorical variables and Wilcoxon rank-sum test for continuous variables, which did not adhere to the normal distribution. The association between treatment with empiric penicillin-G/V monotherapy and mortality was evaluated with logistic regression analysis and adjusted for potential confounders (CURB65, number of comorbidities, admission to the ICU and age). Missing data are accounted for in Table 1 and only patients with complete data on outcome and potential confounders were included in the adjusted analysis.

**Table 1:** Patient characteristics on admission

Patient characteristics	Study population
	*N* = 1320
Age, median (IQR)	71 (58–81)
Gender, male	47% (626)
Nursing home, Yes	11% (145)
Co-morbidities	
COPD	19% (243)
Asthma	8% (105)
Other chronic respiratory disease^a^	4% (49)
Malignancy	9% (119)
Chronic heart disease	24% (320)
Chronic liver disease	1% (12)
Chronic kidney failure	3% (41)
Chronic neurological disease	14% (188)
Diabetes	12% (162)
Number of comorbidities	
One	35% (466)
More than one	25% (325)
Biochemistry, median (IQR)	
CRP, mg/l	108 (47–214)
White blood cell count, x 10^9^/l	12.2 (9.2–15.7)
Urea, mmol/l	6 (4–9)
Vital parameters on admission, median (IQR)	
Diastolic blood pressure, mmHg	75 (65–85)
Systolic blood pressure, mmHg	133 (119–149)
Pulse rate,/min	94 (81–106)
Respiratory rate,/min	20 (16–24)
Temperature, °C	37.7 (37.0–38.5)
Oxygen therapy	58% (744)
Findings on chest x-ray	
Multilobar infiltrate	31% (403)
Risk assessment	
CURB-65: 0–1	53% (605)
CURB-65: 2	29% (330)
CURB-65: 3–5	18% (202)
Antibiotic treatment^b^	
Penicillin-G/V monotherapy^c^	45% (590)
Penicillin-G/V in combination^d^	6% (74)
Macrolide monotherapy	3% (36)
Other beta-lactam monotherapy^e^	30% (396)
Other beta-lactam in combination^d^	10% (132)
Other	7% (92)

Data are presented as % (counts), unless otherwise indicated

*COPD* Chronic obstructive pulmonary disease, *CRP* C-reactive protein

^a^bronchiectasis, pulmonary fibrosis, sarcoidosis, sleep apnoea and pulmonary cancer. All variables had less than 5% missing except smoking status (294 (22%)) and respiratory rate (144 (11%))

^b^Empiric therapy

^c^Recommended dose: 2 mio units x 3 daily adjusted according to weight and renal function

^d^Preferably macrolide or quinolone

^e^Preferable Cefuroxime. Recommended dose: 1500 mg x 3 daily adjusted to weight and renal function

All *p*-values were two-sided and a *p*-value of <0.05 was regarded statistically significant.

We calculated the incidence of CAP only for 2011 by dividing the number of new cases by the number of persons at risk (inhabitants 18 years or older).

We used SAS for Windows statistical software, version 7.1 (SAS Institute, Inc., Carey, NC).

## Results

**Fig. 1:**
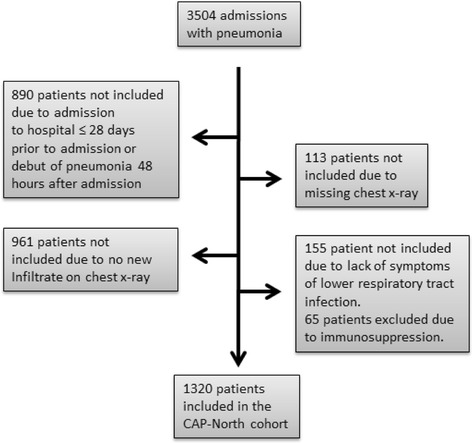
Screening of patients for inclusion

Overall 3504 patients were hospitalized and diagnosed with pneumonia during the 18 months study-period (Fig. 1). The majority of patients were excluded because they did not have an infiltrate on the chest x-ray or they had a nosocomial infection. Ultimately 1320 patients were included in this study and constitute the CAP-North cohort.

**Fig. 2:**
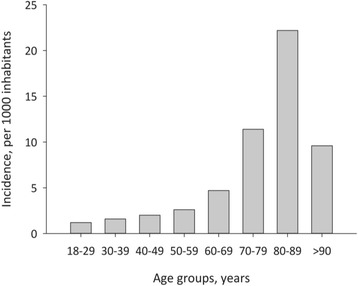
The incidence of community-acquired pneumonia

The incidence of hospitalized CAP, calculated on the basis of the population in the region, was 3.1:1000, increasing with age to 8.9:1000 in patients > 65 years and 14.7:1000 in patients >75 years (Fig. 2).

### Baseline characteristics

Patient characteristics on admission are shown in Table 1. The median age was 71 years (IQR; 58–81), 11% were nursing-home residents and 60% had one or more co-morbidities. One or more chronic respiratory conditions were present in 363 (28%) patients. The majority of patients (53%) presented with mild (CURB-65: 0–1) and moderate (29%) (CURB-65: 2) CAP, and 31% had multi-lobular infiltrates on chest x –ray.

### Microbiological findings

Overall, 19% (248/1320) of the patients and 23% (248/1083) of those who had a sample taken, had a pathogen detected. *Streptococcus pneumoniae* (*n* = 67) was the most common followed by *Haemophilus influenza* (*n* = 57), and *Mycoplasma pneumoniae* (*n* = 36) (Table 2).

**Table 2:** Aetiology by full study population, by all microbiological tested and of pathogen specific testing

	Study population	Microbiologically tested	Pathogen specific tests
	*N* = 1320	*N* = 1083	
Pathogen			
*Streptococcus pneumoniae*	67 (5.1%)	67 (6.2%)	67/1079 (6%)^a^
*Haemophilus Influenzae*	57 (4.3%)	57 (5.3%)	57/1075 (5%)^b^
*Mycoplasma pneumoniae*	36 (2.7%)	36 (3.3%)	36/213 (17%)^c^
*Moraxella catarrhalis*	21 (1.6%)	21 (2.0%)	21/1075 (2%)^b^
*Staphylococcus aureus*	12 (0.9%)	12 (1.1%)	12/1075 (1%)^b^
*Pseudomonas aeruginosa*	11 (0.8%)	11 (1.0%)	11/1075 (1%)^b^
*Escherichia coli*	11 (0.8%)	11 (1.0%)	11/1075 (1%)^b^
*Legionella pneumophilia*	5 (0.4%)	5 (0.5%)	5/288 (1.7%)^d^
Others	28 (2.1%)	33 (3.0%)	33/1083 (3%)

Data are presented as counts (%)

^a^Pathogen detected by microscopy, culture of sputum or blood or urinary test for *Streptococcus pneumoniae*

^b^Pathogen detected by microscopy, culture of sputum or blood

^c^Pathogen detected by PCR analysis

^d^Pathogen detected by PCR analysis or urinary antigen test for *Legionella pneumophilia*

Blood cultures were performed in 74% of the patients and microscopy and culture of sputum samples in 38.5% (Table 3). 66 patients had bacteraemia. In patients diagnosed with *Streptococcus pneumonia* 60% (40/67) suffered from bacteraemia, while this only applied for 5% (3/57) of patients diagnosed with *Haemophilus Influenzae*. Microbiological testing was not performed in 237 (18%) patients.

**Table 3:** Microbiological findings in different sample types

	Tested n(%)	Positive *n* (%^a^)	Three most common pathogens, *n* (%^b^)
	*N* = 1320		
Blood, (microscopy/culture)	981 (74%)	66 (7%)	*Streptococcus pneumoniae,* 40 (61%)
			*Staphylococcus areus,* 4 (6%)
			*Escheria coli,* 4 (6%)
Sputum/tracheobronchial secretion, (microscopy/culture)	501 (38%)	149 (30%)	*Haemophilus influenza,* 56 (36%)
			*Streptococcus pneumonia,* 27 (18%)
			*Moraxella catarrhalis,* 21 (14%)
Test for atypical bacteria, (PCR analysis)	213 (16%)	41 (19%)	*Mycoplasma pneumoniae,* 35 (85%)
			*Legionella pneumophilia,* 3 (7%)
			*Chlamydia pneumoniae,* 2 (5%)
Urine antigen test	133 (10%)	7 (3%)	*Streptococcus pneumoniae,* 4 (57%)
			*Legionella pneumophilia,* 3 (43%)

^a^Percentage of tested

^b^Percentage of positive tests

### Empiric antibiotic treatment and clinical outcome

Penicillin-G/V was the most frequently used antibiotic, mainly as mono-therapy (44.7%) or in combination (5.6%), and 63 (4.8%) received the treatment orally. Cephalosporin’s were used as monotherapy in 25.5% of patients. In total, 77% of the patients initially received monotherapy (Table 1).

The median LOAB was 10 days (IQR 8–12) and 70% of the patients were treated for more than 8 days. The median LOIVAB was 3 days (IQR 2–6). Patients were admitted to the hospital for a median of 5 days (IQR 3–8) (Table 4).

**Table 4:** The course of community-acquired pneumonia

Outcome	Study population
	*N* = 1320
Treatment duration, median days (IQR)	
IV antibiotic^a^	3 (2–6)
Total antibiotic^b^	10 (8–12)
Length of stay	5 (3–8)
Complications	
Any complication	21% (277)
Co-infection	14% (182)
Pneumonia associated^c^	6% (79)
Renal failure	1% (15)
Stroke	1% (12)
Acute myocardial infarction	3% (34)
ICU admission	9.6% (127)
Mortality	
In-Hospital	8% (111)
30 days	11% (149)
90 days	15% (203)
180 days	19% (246)

Data are presented as % (counts), unless otherwise indicated

*ICU* Intensive care unit, *IV* Intravenous

All variables have less than 5% missing

^a^Range: 0–95 days

^b^Range: 0–97 days

^c^empyema, including pleural effusion treated as empyema, and lung-abscess

Complications, severe outcome, and mortality were assessed and we found that 270 (21%) had one or more complications, 127 (9.6%) patients were admitted to the ICU, and 111 (8%) died within the hospital. Mortality on days 30, 90 and 180 was 11, 15 and 19% respectively (Table 4).

### Empiric treatment with small-spectrum penicillin

Patients treated with penicillin-G/V monotherapy had a lower CURB-65 score, less co-morbidities, fewer admissions to the ICU and a lower mortality in the unadjusted analysis (Table 5). We found no association between penicillin-G/V monotherapy and mortality after adjusting for confounders: CURB-65 score, number of co-morbidities (0, 1 or >1), admission to the ICU, and age (Table 6).

**Table 5:** Comparison of patients by antibiotic treatment: penicillin-G/V monotherapy versus broad-spectrum antibiotics

	Monotherapy penicillin-G/V	Other than penicillin-G/V monotherapy	*p*-value
	*n* = 590	*n* = 725	
Age, median (IQR)	70.5 (57–81)	72 (59–82)	0.30^b^
CURB-65			
0–1	58.8% (295/502)	48.6% (306/630)	<0.0001^a^
2	29.1% (146/502)	29.1% (183/630)	
3–5	12.1% (61/502)	22.3% (141/630)	
Co-morbidities			
0	45.1% (264/585)	35.1% (252/718)	0.001^a^
1	32.8% (192/585)	37.7% (271/718)	
> 1	22.1% (129/585)	27.2% (194/718)	
ICU admission	6.1% (36/590)	12.6% (91/725)	<0.0001^a^
In-hospital mortality rate	6.4% (38/590)	10.1% (73/725)	0.02^a^
90 day mortality rate	11.7% (69/590)	18.5% (134/720)	<0.0009^a^

Data are presented as % (counts), unless otherwise indicated

^a^Chi-square

^b^Wilcoxon rank sum

**Table 6:** Risk of death in patients treated with penicillin-G/V monotherapy

	Univariate		Multivariate^a^	
	OR (CI)	*P*-value	OR (CI)	*P*-value
In hospital mortality				
Penicillin-G/V monotherapy	0.62 (0.41–0.93)	0.02	0.92 (0.55–1.53)	0.74
broad-spectrum (ref)	1	-	1	-
90 day mortality				
Penicillin-G/V monotherapy	0.58 (0.43–0.80)	0.0008	0.77 (0.52–1.14)	0.19
broad spectrum (ref)	1	-	1	-

^a^Adjusted for CURB-65, number of co-morbidities (0, 1 or >1), admission to an intensive care unit (Y/N) and age

*OR (CI)* odds ratio with 95% confidence interval. Cases used in the adjusted analysis: 1122

A subgroup analysis of the patients treated with penicillin-G/V monotherapy showed that patients who died in-hospital compared with survivors were older, 81 (IQR; 77–89) versus 70 (IQR; 56–80) years of age (*p* < 0.0001) and more often nursing home residents, 32% versus 6% (*p* < 0.0001). Further, they had more comorbidities, 76% versus 53% had ≥ 1 comorbidity (*p* = 0.002) and a higher CURB-65 score, 90% versus 38% had CURB-65 ≥ 2 (*p* < 0.0001). We found similar results when looking at 90-days mortality (data not shown). Of the 38 patients who received monotherapy with penicillin G/V and died in-hospital, 71% had an unknown aetiology. Of the remaining 11 patients, 3 were infected with Streptococcus spp. susceptible to penicillin G/V, 3 with *Klebsiella pneumonia*, 3 with *Escherichia coli*, 1 with *Haemophilus influenza,* and 1 with *Mo*raxella catarrhalis. Of the 8 patients with pathogens non-susceptible to penicillin, 3 had a CURB65 score of more than 2 and monotherapy with penicillin was therefore an incorrect choice of empiric antibiotic according to guidelines. One had an unknown CURB-65 score due to missing data.

We addressed whether in-hospital mortality in the different CURB-65 categories was related to choice of empiric treatment and we found that the in-hospital mortality for patients treated with penicillin-G/V monotherapy with a CURB-65 score ≤ 2 was 4.3% and not different from 5.9% for patients treated with other than penicillin-G/V monotherapy (*p* = 0.26). Sixty-one patients received empiric treatment with monotherapy penicillin-G/V despite of having a CURB-65 score ≥ 3, and the mortality was 19.7% compared to 19.9% for patients treated with other than penicillin-G/V monotherapy (*p* = 0.98).

## Discussion

The incidence of hospitalized CAP was 3.1:1000 adult inhabitants in the Danish region of North Zealand. The incidence increased substantially with age. Nearly half of the patients were treated empirically with penicillin-G/V and the median duration of antibiotic treatment was 10 days. Patients treated with penicillin-G/V had less severe pneumonia than patients treated with broad-spectrum antibiotics or combination therapy and penicillin monotherapy was not associated with increased mortality after adjusting for CURB-65, co-morbidities, admission the ICU and age.

The incidence is comparable to other studies from the UK, USA and Germany reporting incidences of hospitalized CAP of 1.1, 2.5 and 3.0 per 1000 respectively [12–14].

Microbiological sampling was not systematically performed; 18% did not have any microbiological samples taken and a pathogen was detected in only 19% of all the patients. This low yield does not differ from most other studies. Even in prospective studies systematically collecting samples, the diagnostic yield is low. In a recent study only 38% of the patient had a confirmed microbiological diagnosis [13]. In contrast to most other studies however, the empiric therapy was much more restrictive; monotherapy was administered to 77% of the patients and 45% received penicillin-G/V alone. Penicillin-G/V monotherapy is rarely used in southern European countries and the USA [15]. In a European observational multicentre study, 50% of adults hospitalized with CAP received monotherapy but none received penicillin-G/V [16]. Meanwhile, empiric treatment with β-lactam as monotherapy was given to 63% of the patients in a Dutch study [6].

The ERS guidelines for treatment of hospitalized CAP do not recommend penicillin as monotherapy [7] whereas, Danish guidelines during the current study recommended benzylpencillin as monotherapy for patients with a CURB-65/CRB-65 score < 3, and combination therapy with quinolones or macrolides for CURB-65 ≥ 3 (http://www.infmed.dk/guidelines). This is in line with guidelines from other Scandinavian countries and the Netherlands [5, 6]. The differences in recommendations are due to a low penicillin resistance of *Streptococcus pneumonia* in Denmark of < 1% whereas it is, e.g., in Spain > 10% (http://ecdc.europa.eu/en/healthtopics/antimicrobial_resistance/database/Pages/map_reports) and a very conservative antibiotic policy encouraging small spectrum antibiotic.

In our study, patients treated with penicillin-G/V had less severe CAP. There were no differences in mortality associated with the use of penicillin-G/V or broader-spectrum antibiotics in the adjusted analysis, similarly to the findings in a Danish study from 2001 [17]. Because penicillin monotherapy was given to patients with mild disease (CURB-65 < 3), mortality is expected to be low independently of the treatment and we cannot conclude whether mortality would have been lower if broad spectrum antibiotic or combination therapy were given. Mortality in those with CURB-65 ≤ 2 was still substantial in both treatment groups (4.3–5.9% respectively) indicating that mortality is not solely related to initial severity assessment, but also to comorbidities and complications during admission [18, 19].

Patients who were treated with empiric penicillin-G/V monotherapy and died, were older, had more co-morbidities and a higher CURB-65 score than survivors. Penicillin-G/V monotherapy may have been insufficient in these patients and apparently guidelines were not followed in some cases. Pathogens non-susceptible to penicillin were found in 4 patients who were treated with penicillin-G/V monotherapy, had a CURB-65 score less than 3 and died. Although guidelines were followed for these patients, they received empirical treatment not matching their causative pathogen. However, due to the retrospective design, we cannot conclude if the poor outcome was due to monotherapy with penicillin and if outcome would have been better with another antibiotic agent.

Overall, penicillin-G/V appears safe in the correct patient group, namely those hospitalized with mild to moderate CAP (CURB-65 < 3) in a setting with a low degree of resistance of *Streptococcus pneumonia* against penicillin.

In our study, the median duration of treatment with antibiotics was 10 days and 70% of patients received more than the maximum 8 days which is recommended by European guidelines for responding patients [7]. Similarly, Reissig et al [20] and the REACH study [16] reported 11 and 10 days respectively. Duration of 10–11 days, however, exceeds recommendations in European and Danish guidelines ([7], http://www.infmed.dk/guidelines). Danish guidelines recommend duration of 10–14 days only for patients with CURB-65 ≥ 3, which can explain extended treatment duration for a smaller fraction of the study population. Due to the retrospective nature, the unsystematic microbiological sampling, and the low proportion of positive microbiological findings, we cannot draw firm conclusion on significant risk factors for prolonged treatment duration.

Recent studies have shown that antibiotic treatment for 3–5 days for mild to moderate CAP does not impair effectiveness or safety [21–23]. There is a need to focus on assessing alternative strategies to reduce antibiotic use in order to minimize the risk of adverse events and antibiotic resistance associated with extended duration of antibiotic therapy.

Comparing patients in the CAP-North cohort with other European CAP cohorts [14, 16, 24, 25] some differences were apparent. Compared with patients from the CAPNETZ cohort [25], our patients were older (mean age 72 versus 66), more frequently women (60% versus 47%), presented with more severe CAP (18% CURB-65 > 2 versus 4% CRB-65 > 2), and had a higher 30 days mortality (11% versus 4.3%). The differences could be due to our inclusion exclusively of hospitalized patients, as well as inclusion of all patients with severe CAP who are often challenging to include in a prospective study.

The major strength of this study is that we were able to identify all patients hospitalized and diagnosed with pneumonia in our region, due to our complete regional dataset. Our design did however not allow us to account for patients with CAP who were given an inaccurate ICD-10 code. We ensured that only patients with CAP were included by reviewing every patient-file for symptoms on admission, hospitalization within the last 28 days, and chest-x-ray for infiltrates. Moreover, the three hospitals cover both urban and rural areas of the capital region in Denmark and thus we believe our cohort to be representative of patients hospitalized with CAP in developed countries. Thus, our cohort represents a “real-life” population of patients hospitalized with CAP.

The main limitation of this study is the retrospective design, which made us dependent on the data recorded in the patient files and the results should be interpreted accordingly.

The cohort consists of a heterogeneous population, specifically patients who received treatment with monotherapy penicillin G/V were less sick, than patient who received broader spectrum antibiotics, according to the CURB65-score. We adjusted for this in the regression analysis, but we cannot exclude that residual confounding exists.

We were not able to account for changes in antibiotic therapy after initial assessment, i.e. due to complications or microbiological findings. Further, we could not account for the exact dosages prescribed but only recommended dosages. Furthermore, treatment duration varied among patients.

Approximately 50% of patients had a CURB-65 score of 0–1. It is suggested that CAP patients with a CURB-65 score of 0–1 can be treated as outpatients, albeit CURB-65 has several limitations and should not be the sole basis for deciding treatment allocation [26–28].

## Conclusion

The incidence of patients hospitalized with CAP was 3.1:1000. Nearly half of the patients were treated with empiric penicillin-G/V monotherapy without an increase in mortality. Treatment duration exceeded recent guidelines. Our results are in agreement with the current conservative antibiotic strategy outlined in Danish guidelines, recommending penicillin treatment.

It is important to ensure compliance with guidelines concerning treatment duration and validation of strategies to reduce duration of treatment in order to minimize unnecessary side-effects. Finally prospective, randomized control trials would be necessary to confirm whether empiric monotherapy with penicillin-G/V is recommendable.

## Abbreviations

CAP: Community-acquired pneumonia
ICU: Intensive care unit
LOAB: Length of antibiotic treatment
LOIVAB: Length of intravenous antibiotic treatment
LOS: Length of stay
LRTI: Lower respiratory tract infection.
